# Urban Agriculture as a Means to Food Sovereignty? A Case Study of Baltimore City Residents

**DOI:** 10.3390/ijerph191912752

**Published:** 2022-10-05

**Authors:** Brionna Colson-Fearon, H. Shellae Versey

**Affiliations:** Department of Psychology, Fordham University, 441 E Fordham Rd, Dealy Hall 226, Bronx, NY 10458, USA

**Keywords:** alternative food networks, food insecurity, food access, urban agriculture, community farming

## Abstract

A large body of research suggests that neighborhood disparities in food access persist. Emerging evidence suggests that the global COVID-19 pandemic likely exacerbated disparities in food access. Given the potential role that alternative food networks (AFNs) and local food sources may play during times of extreme scarcity, this study examines urban agriculture (e.g., community farms and gardens) as a sustainable strategy to address food insecurity. In-depth qualitative interviews with fifteen community stakeholders revealed several major themes including food insecurity as a feature of systemic racism, food affordability and distance to food as major barriers to food security, and the role of AFNs in creating community empowerment. Our findings indicate that urban agricultural practices help build social capital, inform and educate community members about healthy eating behaviors, and facilitate the distribution of affordable food. Implications for future research and policy targeting sustainable food distribution in marginalized communities of color are discussed.

## 1. Introduction

Variability in the way that food is produced, sold, and consumed leads to significant food inequities. On a neighborhood level, studies find significant disparities in neighborhood food environments, specifically among the types of food outlets available, food products offered, and dietary intake [1,2,3,4,5,6]. In the United States, it is well documented that lower-income and African American neighborhoods, for example, have fewer supermarkets, more liquor stores, and more convenience stores than higher-income and White neighborhoods, respectively [7,8]. Even after adjusting for store type, there are fewer healthy food options and lower quality foods in low-income and African American neighborhoods [9,10,11]. This literature indicates that there is significant variation in food access across neighborhood types tied to larger systems; namely spatial racism and discrimination, creating barriers to healthy eating for lower-income residents and people living in predominantly African American neighborhoods [12].

Studies examining food access disparities have primarily focused on documenting food deserts, with less attention paid to solutions that may alleviate them. Emerging literature suggests that alternative food networks (AFNs), systems that directly connect local producers (e.g., farmers) with distribution channels and consumers, may help reimagine consumers’ spatial relationship with food, especially in areas where access to fresh food is low. In fact, studies find that AFNs not only improve food security, but also address issues such as unemployment, community decline, and food deserts [13]. To date, much of the research addressing food deserts relies on traditional food system solutions, such as the introduction of large supermarkets into communities. Yet, findings indicate that the success of supermarket interventions has been mixed, suggesting the need for more comprehensive and sustainable food source alternatives for residents living in communities with fewer options. Previously, Cummins, Flint, and Matthews found that opening a new supermarket in a Philadelphia neighborhood considered a “food desert” moderately improved perceptions of food accessibility; however, there was no significant change in residents’ body mass index (BMI) or fruit and vegetable intake, suggesting that supermarkets alone are insufficient to change eating behaviors [14]. Similarly, Ghosh-Dastidar and colleagues found that introducing a new supermarket into a low food-access neighborhood did not result in more healthy food availability than would have been expected otherwise [15]. 

The current paper examines urban agriculture within a metropolitan area described as a ”food desert” to better understand the role urban gardens and urban farms play in lower-access cities and neighborhoods. We begin with a review of theoretical perspectives characterizing food environments, and then examine the importance of urban agriculture (as one form of AFN) in Baltimore, Maryland, where approximately one in four city residents are food insecure [16].

### 1.1. Theoretical Considerations

#### 1.1.1. Food Deserts, Food Apartheid, and Food Sovereignty

Most literature on low-food access neighborhoods overwhelmingly focuses on describing, measuring, and characterizing “food deserts”, an *outcome* of a political economic system that structures access to food spatially (e.g., by where you live) [7,8,17,18,19,20]. While initially conceptualized as a metaphor to describe areas where access to healthful food is limited, a persistent focus on food deserts obscures the macro-level drivers that create food deserts in the first place [21]. In other words, while the term is commonly used by researchers, it does not adequately or accurately reflect *how* and *why* areas where people have lower incomes and lower access to healthy food become that way. This misnaming is an important oversight because the language used to describe people, processes, and outcomes is important and shapes strategies for systemic change [22,23,24].

Developed by food justice advocate, Karen Washington (2018), the term “food apartheid” describes the broad framework that creates inequitable food environments. According to the National Black Food & Justice Alliance (http://www.blackfoodjustice.org (accessed on 10 June 2022)), food apartheid is “the systematic destruction of Black self-determination to control one’s food, the hyper-saturation of destructive foods and predatory marketing, and the blatantly discriminatory corporate-controlled food system that results in (communities of color) suffering from some of the highest rates of heart disease and diabetes of all time”. Therefore, food deserts do not simply emerge organically; they are the product of deliberate, exclusionary practices and policies that restrict food access to lower-income (and predominantly Black and Latino/x) neighborhoods. This exclusion is facilitated, in part, by a traditional food supply chain that distances residents from their food. Despite calls to reframe food deserts as an outcome (rather than a cause) of structural racism [12], research has been slow to realign its focus to the large-scale drivers of food inequity [25] and the equitable solutions that might overcome these drivers.

One solution that has been proposed to address food apartheid is a shift towards local, community-owned and operated food networks (i.e., food sovereignty). Food sovereignty describes “the right of local people to control their own food systems, including markets, ecological resources, food cultures, and production modes” [26]. Food sovereignty is an ongoing process in which necessary changes are pursued to achieve the state of having food security [27,28,29]. Research on food sovereignty measures is limited within the academic literature. However, supporting AFNs broadly, and urban agriculture specifically, has been proposed as one pathway to achieving food sovereignty.

#### 1.1.2. Alternative Food Network Solutions

Research suggests that reframing and reconsidering how food is supplied to communities is a potentially more fruitful strategy to lessen disparities across food environments [16,28,30,31,32,33]. “AFNs” is a broad term that refers to local food producers and distributors that facilitate more direct food access to consumers, which can include community gardens, urban/rural farms, farming cooperatives, farm-to-school programs, and farmer’s markets [34,35]. While some efforts in this area have been criticized for not being fully inclusive of lower-income groups, other research suggests an alignment between alternative food systems and food sovereignty values – cooperation, self-determination, and food justice [36,37,38]. For example, the Growing Food and Justice Initiative (GFJI) maintains that food insecurity is a form of systematic racism; therefore, designing sustainable and equitable food systems necessarily involves dismantling racism. These and other models of cooperative farming (used for years across various contexts) may provide a more effective and sustainable method to providing quality, health-promoting foods in low-access areas [39,40]. One popular cooperative farming model is community-supported agriculture (CSA), a system where consumers (also called members) pay a small fee to a local farm in exchange for fresh and healthy produce. Among members, CSAs have been found to increase fruit and vegetable consumption and improve household food environment [41]. Though similar, urban/rural farms and gardens that operate within so-called food deserts are not well-studied. Part of this discordance is due to a lack of scholarship about community-run producer models, and to some extent, a de-prioritization of local community knowledge (i.e., community residents as experts in their own experiences in relation to food systems), distorting perceptions of what food access actually looks like in urban food environments [42,43,44,45].

### 1.2. Current Study

Generally, research finds that a lack of high-quality, reasonably priced, healthy foods, and reliable transportation are barriers to healthy eating in low-access markets [46,47,48,49,50,51,52,53,54,55]. However, few of these studies have examined the potential utility of urban agricultural markets in filling this gap [46,51,52,55]. Furthermore, economic variability and recent events, including the global COVID-19 pandemic, have led to greater challenges in food security, particularly for marginalized and lower-income groups [56]. Since these issues are likely to be ongoing, understanding how proximity to alternative food outlets, such as community gardens and urban farms, may alleviate hunger is important for informing future research and policy interventions.

This paper uses a qualitative approach to explore the role of urban agriculture in Baltimore, Maryland during the COVID-19 pandemic. In a series of interviews with local urban farmers, food advocates, city residents (i.e., food equity advisors), and academic scholars, we examined urban farming cooperatives as “alternative food systems” to better understand the potential these sources may hold for addressing food security in low-access neighborhoods, particularly during times of extreme emergencies and scarcity. One way to achieve food sovereignty is to build on the experiences, practices, and values of community members by engaging their expertise about what food and food access means in their lives [57]. Therefore, the guiding research question for this study was: What role does urban agriculture play in facilitating food access in Baltimore City?

## 2. Materials and Methods

### 2.1. Study Area: Baltimore, MD, as a Case Study

Eighty-six percent of Baltimore residents live in a limited-supermarket access (LSA) area, meaning that residents within LSA areas travel farther to reach supermarkets than residents of non-LSA areas. Additionally, the city is ranked first as an area in need of intervention(s) to ensure greater food access equity [58]. Baltimore is the largest city in the state of Maryland, with a population of 585,708 as of April 2020; 62% of residents are Black/African American and 27.5% of residents are non-Hispanic whites, the second largest racial group [59]. The city’s poverty rate (20.0%) is higher than the national average (11.4%) and the median household income is $52,164 (USD), nearly $15,000 less than the United States average of USD $67,521 [59,60]. Approximately 57,500 Baltimore households receive assistance from the Supplemental Nutrition Assistance Program (SNAP)—24% of the total population [61]. One in three high school students in Baltimore City is either obese or overweight, and less than half eat at least one serving of vegetables a day [62]. 

### 2.2. Participants and Recruitment

Participants were recruited through direct outreach, internet recruitment (i.e., email, social media posts), and snowball sampling. The qualitative framing of this study (e.g., phenomenology) determined the sampling approach. For example, phenomenological research recommends recruiting a sample of individuals that have experienced the phenomenon under inquiry [63]. Therefore, following initial recruitment, criterion sampling was applied to ensure that all study participants had some experience with (or close knowledge of) the urban agricultural system in Baltimore [63].

The authors identified and contacted local activists, advocates, and scholars involved in Baltimore food justice issues (the central topic of interest), and then invited participants to schedule an in-depth interview. Additional inclusion criteria required individuals to: (1) live/work in urban agriculture in Baltimore; or (2) conduct food justice work in the area. Researchers identified participants across a range of sectors (e.g., community members, academic researchers, and government officials). Fifteen individuals identifying as urban farmers, community residents, leading academic scholars, or food equity advisors participated in the study. Food equity advisors are Baltimore residents who work collaboratively with the city’s Department of Planning to create an equitable community food system. As with all research, the participants in this study were volunteers; therefore, the representativeness and proportionality to the source community may not be exact.

All participants were over the age of 18, and either lived in Baltimore or conducted food justice work in the city. Nine were urban farmers, three were food equity advisors, two were academic scholars, and one was a former employee of the Baltimore City Health Department. Nine participants identified as women and six identified as men. Ten participants identified as Black/African American (*n* = 10) and five identified as white (*n* = 5). 

After obtaining informed consent, participants participated in semi-structured qualitative interviews virtually. All interviews were conducted between June 2020 and February 2021 by both authors. Due to the timing of this study and COVID-19 travel restrictions, nearly all interviews were conducted via phone or online conferencing platform. Participants were compensated with USD $30.00 for their participation. 

### 2.3. Interviews

An interview protocol was developed by both authors to understand the role of local urban agriculture and food access in the Baltimore city area. Interview questions asked about definitions of the term “food desert,” perceptions of local food access, and the perceived role of urban farming in increasing food access to Baltimore community residents. The interview approach was phenomenological and relational [64,65], meaning that researchers built a rapport with participants to understand the phenomenon under study, as described by respondents with close knowledge about the topic. In other words, participants were considered privileged sources of information. Thus, emphasis was placed on understanding urban agriculture *during* the pandemic *within* a Baltimore context rather than eliciting right or wrong answers, or achieving generalizability [66].

The authors followed an inductive analysis approach to preserve the validity of the data, and highlight the scope of food access inequality that participants either experienced or observed as a consequence of COVID-19 specifically, and generally across Baltimore [67,68]. Interviews were structured to center participants’ narratives, including learning about how they made meaning of their experiences rather than imposing a particular interpretation (i.e., by the researcher) [69].

### 2.4. Coding and Data Analysis

All interviews were read and coded by both authors. The researchers (BCF and HSV) reviewed the audio and written transcriptions of the data, and recorded field notes. The authors then used an open-ended and iterative approach to coding [70,71]. Codes were developed from text extracts, discussed, classified, organized into a codebook, and later grouped into themes using Braun and Clarke’s (2006) six-phase thematic approach [71].

Inductive thematic analysis was used because of its flexibility in a variety of theoretical frameworks. Initial coding agreement was relatively high (κ = 89%) between the two coders; disagreements about codes were discussed, resolved, and then re-coded for subsequent rounds to establish consistency. We employed a combination “unit and focus analysis” coding scheme, identifying recurring, major themes [72]. The four major themes that emerged were: (1) food access inequality; (2) community empowerment; (3) health promotion; and (4) environmental sustainability. Block quotes framing themes were identified and extracted to characterize perceptions of food accessibility. To ensure participant confidentiality, all names reported in the manuscript are pseudonyms.

### 2.5. Methodological Integrity

During data collection, interviewers recorded field notes on participants’ responses and asked for clarification when necessary. These notes were referenced during coding and data analysis to ensure accuracy. Researchers were also reflexive throughout, noting personal connections to the subject matter and the ways in which this guided analysis. The authors participated in debriefing sessions, discussing their own subjectivity in relation to the study, as well as the best practices to ensure all findings were based within the data.

## 3. Results

Findings revealed that food access remains a challenge in Baltimore; a challenge that was exacerbated due to the pandemic. Participants expressed four main themes in relation to the perceived role of urban agriculture in facilitating food access and, to a larger extent, food sovereignty. If supported, urban agriculture could: (1) address food access inequality; (2) support community empowerment; (3) encourage health promotion; and (4) provide sustainable access to healthy food.

### 3.1. Food Access Inequality

The most frequently mentioned theme across interviews was food access inequality. Food access inequality was coded according to conventional definitions found in the literature; participants mentioned inequality resulting from a sustained lack of resources devoted to food equity across Baltimore—disparities that worsened during the COVID-19 pandemic. Therefore, in describing the role that urban agriculture plays in Baltimore City, most participants discussed local farming and agriculture as an underexamined and under-resourced equity strategy. Within this broad theme, three subthemes emerged: systematic racism, affordability, and distance to healthy food.

#### 3.1.1. Systematic Racism

When presented with prompts about why such stark food disparities exist across neighborhood lines, several participants highlighted systematic racism as a primary driver. For example, in response to the question, “What do you see as the primary challenge(s) to food justice in Baltimore City?” Saundra, an academic researcher and Baltimore resident, attributed differences in food access to long-standing systematic issues that have contributed to neighborhood disparities.

We didn’t just wake up one day and arrive at food insecurity, income insecurity, no jobs, and a lack of access to quality food…we have the intersection of quite a few pandemics at one time now. But the *real* pandemic is American apartheid, segregation, inequality, and racism. America has been in crisis for 400 years.

(Saundra, Academic)

Drawing on her experiences as a Baltimore native and public health researcher, Saundra discusses how the current food system, rooted in neighborhood segregation, deprives communities of the right to healthy food. These culminating crises, though made visible by the pandemic, are not new. In relating disparities to potential solutions, Saundra emphasizes the role of structural solutions (e.g., community co-ownership of land) to correct the legacy of structural racism in creating low food access for Baltimore residents.

Rodney, another academic researcher, describes how racism informed the planning and design of Baltimore City. According to him, “Black and brown communities were intentionally pushed” to the edges of society, where they lacked access to several needs, including quality food.

The planning of the city was no accident …in the 1880s, it was one of the first cities in the U.S. to do really explicit racialized zoning, and the Baltimore today that we have is a result of that…We were complicit, as urban planners, in redlining. The way our cities look is a direct result of urban planning. Not to say urban planners are all racist, but it is to say that we have been using a toolkit specifically designed by the white supremacist regime to segregate, to separate, to discriminate, to differentiate.

(Rodney, Academic)

Rodney highlights that the design of disparate neighborhoods that led to modern-day food deserts, were the result of decisions made to support residential segregation by race and class. This process is the foundation of racial spatialization and restricted access to basic resources, including food.

Reinforcing the idea that low-access food areas are the result of racist toolkits and policies, Rosslyn discusses how segregation impacts not only food, but also the health and psychological well-being of Black city residents.

Food apartheid is intentional violence—physical, emotional, mental violence towards Black and brown people…it makes it hard for people to eat healthy produce, to grow healthy produce, and to be paid a living wage for their work.

(Rosslyn, Farmer)

The idea that a lack of access to food is a form of violence clearly outlines the drivers (e.g., systemic racism) and consequences of disparate access. The inability to grow and eat healthy food also aligns with a systems view of health and health disparities that food impacts other areas of life. Rosslyn notes that urban agriculture can disrupt this process; improving health through food that residents can grow themselves enables greater control over the foods they eat, and in turn, their health. During the height of the COVID-19 pandemic, for example, Rosslyn mentioned that many residents turned to community pantries and churches for food relief, given the difficulties in purchasing food at traditional supermarkets. Having limited control over food access meant primarily relying on temporary safety nets, such as charitable groups and community generosity.

Monica, a Baltimore resident (and Food Equity Advisor), also discusses the role of racial and economic determinants in shaping food access in Baltimore specifically, noting a lack of diversity among local food providers in certain neighborhoods.

What attracts a supermarket in the area…is the income that people have…and if there’s other businesses around. And…in Baltimore, if there’s a high concentration of Black people, there’s really not that many businesses; there’s only corner stores, there’s only liquor stores, and it doesn’t really attract any businesses but those.

(Monica, Food Equity Advisor)

Similar to Rodney and Saundra’s emphasis on the role of structural drivers, Monica’s reflection suggests why an overreliance on supermarkets is likely to be an ineffective strategy. Large-scale food providers are less likely to open and operate in lower-income neighborhoods, creating a system in which community residents must rely on external forces to access food. Most participants discussed this as an unsustainable option, noting that an overreliance on supermarkets is a primary factor in maintaining food deserts, whereas urban agriculture is one way to begin to redress these issues, by relying on local options to provide food.

#### 3.1.2. Affordability

The second subtheme, affordability, was also mentioned as a major barrier to accessing fresh and healthy food. SNAP benefits (e.g., food stamps) were discussed as important to relieve some financial burden, especially during the pandemic, when SNAP recipients could shop for food at selected online retailers. However, our participants noted that SNAP is not necessarily available to everyone who needs it, and retail outlets that accept SNAP continue to be limited.

There’s only one store [close by] that accepts EBT. There’s three different corner stores on my block, so the one store that does accept EBT has a little bit of fresh produce…they just don’t have the refrigeration.

(Monica, Food Equity Advisor)

Monica points out the limits of relying on corner stores, often the only type of retail outlet that sells food in a neighborhood. While one of her local corner stores does sell fresh food and accepts SNAP, it is often limited in selection given the lack of refrigeration. Because of this, residents who both rely on SNAP benefits and use corner stores as a primary source of food are severely restricted in their food options.

The point about affordability was raised by several participants including Paula, an urban farmer, who believes that the cost of food is a major issue to food security in Baltimore.

The issue remains of…how does a person without financial…resources get food? And learn where [it] really comes from and how to grow it, so they’re not dependent upon fast food?

(Paula, Farmer)

Paula’s response suggests that food cost and education should be aligned in a way that shifts demand away from cheap, highly dense, fast food, and towards more healthy options. The idea that healthy food is more expensive than fast food was raised as a central reason to support local urban agriculture, since farms and gardens usually provide cheaper produce and oftentimes give away free food.

Esther and Sasha, a food equity advisor and Baltimore resident, respectively, further connect the points between food costs, quality, and decisions about food purchasing.

Sometimes there are substandard supermarkets close to where you live, so you go to those substandard supermarkets and you’ll buy food. I’ve been to supermarkets where you walk in and they smell, and I would not buy anything, but then I look around and there’s so many people in there because they have no choice. This is the closest thing to them, they have to buy there; they don’t have anywhere else to go.

(Esther, Food Equity Advisor)

I’ve noticed that a lot of low-income markets are cheaper and more reasonable.…[but they] don’t care about [your] health. And a lot of high-priced markets that have good quality and fresh vegetables…do care about your health…but it’s too expensive. There’s a Whole Foods…[where] a pack of hot dogs, the cheapest is $3.99. And at Price Rite or Save-a-Lot you get them for 99 cents. So if you want quality, you have to go with the high price.

(Sasha, Food Equity Advisor)

Sasha’s comment reflects the tradeoff lower-income residents often must make between price and quality: high quality often comes at a higher price that is sometimes unattainable. In this way, alternative options offered by community-run gardens and farms would address this issue, providing higher quality produce at lower prices.

#### 3.1.3. Distance to Healthy Food

Finally, distance to healthy food emerged as a significant subtheme. In Baltimore, lower-income residents typically live further away from any grocery or retail food outlets–healthy or otherwise. Across all interviews, participants discussed how a lack of transportation (e.g., car ownership) or physical disability can cause difficulty in traveling to a supermarket, particularly when the closest one is over a mile away. For example, when asked, “When did you first realize there were differences in food access in your community and other neighboring areas?” Esther discussed how a sudden change in mobility made her better understand the challenges individuals with disabilities must go through to obtain food.

Up until ten or twelve years ago when I lost my legs, I had a car, so it meant nothing to go here, there, and everywhere to get food…I didn’t think about other people and their hardships until only a couple of years ago…the challenges were different…seeing the senior citizens or people with disabilities trying to navigate to a store, and the store is where? More than a mile away.

(Esther, Food Equity Advisor)

As Esther points out, traveling to supermarkets that are located over a mile away is difficult for many people, including older adults with mobility limitations and/or those with disabilities. In addressing this point, Tyler, a Baltimore City urban farmer, highlights how a community garden located a few blocks away can provide closer access to food:

Where our farm is [located], the closest supermarket is at least a mile, maybe two or three. So…an elderly person is taking two buses…it’s their whole day to get to a supermarket. Or they’re paying someone an exorbitant amount of money out of their budget…to go pick up stuff because they can’t. It’s inconvenient for them to get to the store.

(Tyler, Farmer)

The burden of transportation adds context to the notion of “access.” Whereas driving to a supermarket may make living in a low-access area easier, few families have that option. According to several participants, taking multiple buses to get to a supermarket is a necessity, “yet extremely time consuming”.

One of Tyler’s motivations in starting a local farm was to address this issue; he saw a need for a healthy food source in the low-access neighborhood where his farm is located. The farm offers fresh seasonal food to residents on a sliding scale to maintain affordability. Community residents volunteer at the farm; however, Tyler and his operational staff run the farm year-round. There is a need for additional farms and gardens in the area. This need presents an additional challenge, since the availability of land is limited, and barriers to owning land within the city limits are high. 

Farmer Jimmy notes that bringing farms closer to residents is essential in reducing the cost of food, directly addressing the issue of food inequality:

Most of Baltimore’s food is imported. It comes from California, Miami, outside of the country and the surrounding counties…so we have to find a way to decentralize the distribution, decentralize the storage, and decentralize the processing. If we are able to do that on our farm, then we’ll be able to create our own food, create our own work, [and] manage our own resources.

(Jimmy, Farmer)

Jimmy raises the point that since most of Baltimore’s food is imported, transportation costs lead to higher food prices for city residents. Growing and selling food locally simplifies some aspects of the food supply chain, making healthful foods less expensive and within reach. All the urban farmers in our study noted that increasing access to fresh, quality food in a low-access neighborhood was their central reason for engaging in urban farming.

### 3.2. Community Empowerment

Urban agriculture was cited as contributing to community empowerment. Community empowerment was coded by mentions of communities taking increased control over systems responsible for food production and distribution. In this way, farms and gardens served a functional need in providing an alternate outlet from which to purchase fresh food. Across multiple interviews, participants discussed the importance of using agricultural knowledge to provide food for their neighbors and teach them about farming methods. Two subthemes emerged: community control and instrumental support.

#### 3.2.1. Community Control

The farmers in our study noted that urban agriculture played a central role in overall community empowerment and self-reliance, providing residents with an opportunity to grow and take care of their own food.

Participants stated that investing in urban agriculture would be a more fruitful and economically sustainable goal than attempting to attract supermarkets into the neighborhood. For example, Esther believes that community-run urban gardens will one day be widespread throughout Baltimore City, and if successful, the barriers of affordability and distance will no longer prevent anyone from accessing fresh fruits and vegetables.

There’s a lot of soil, there’s a lot of abandoned houses in Baltimore City. Tear them down, make a little garden where you plant food, and have the neighbors tend to it, and then when it’s time for harvest, everyone can get it for free. It’s a little far-fetched, but it can happen. If people wanted to eat and eat good, there are vegetables and fruits that we could plant right here in Baltimore that would be successful, but everybody would have to want to come together and participate.

(Esther, Food Equity Advisor)

Esther highlights that limited options can be broadened with urban agriculture, promoting healthier eating by leveraging community and neighborhood resources, rather than relying solely on supermarket solutions. One farmer, Lauren, says she recognized this potential during the start of the pandemic, and is now using land she owns to feed those in her community.

I met this guy who had just…adopted a vacant lot around the corner from my house… and I was like, oh, that’s very cool…I just sort of took over…and now I have a community garden here that I run…With COVID, all of a sudden we had all this time and this property…We said, ”Let’s just go there every day and work and do it and…anybody who wants to come and join and help us grow can.” But also, people are allowed to take food whether or not they help us grow it.

(Lauren, Farmer)

Lauren’s actions speak to the recurring theme that urban farms and gardens are important resources in the community, providing both opportunities for self-sufficiency and community control. In addition to making fresh food affordable and accessible, several respondents discuss strengthening intergenerational community ties as important to maintaining community engagement. For example, Esther highlights the role of children as both teachers and learners of healthy food habits:

There is a program where they go to schools and teach the children how to prepare certain meals with vegetables the kids have probably never even heard of, like zucchini. Some children have never seen what a zucchini looks like, but there is a program where they…teach them how to cook it…then you give them some to take home so they can show their parents how to cook it…teach them while their minds are open and they’re willing to try something new.

(Esther, Food Equity Advisor)

Farmer Jimmy discusses similar outreach his farm does to include younger people, hoping to instill an appreciation for farming.

Urban farms serve as a nucleus in communities when you talk about food security, bringing people together, and…addressing trauma. I really think urban farmers and administrators should go above and beyond by providing support for young people to be involved in urban farming…What we’re doing is we’re creating our own work, managing our own resources, and we are feeding security by growing food with children for everyone in our community.

(Jimmy, Famer)

Jimmy believes that “by teaching children urban farming techniques while they are young, they will grow up understanding their role in providing food for their communities,” perhaps breaking the cycle of food insecurity, which he stated as a primary goal of his work.

#### 3.2.2. Instrumental Support

As a complementary theme to community control, participants spoke about the role of urban agriculture in providing instrumental support, or a tangible solution to accessing affordable, fresh food. Participants spoke about how urban agriculture fills a critical need in the community, providing food to those who need it, and giving away food for those who are unable to afford it. This was noted as especially important during the pandemic. Framing food distribution in this way, as a basic need rather than a commodity, was raised as a central reason why farmers started farming in the community in the first place. Tyler says his farm donates produce to those in need since they often have a surplus:

Our farm partnered with a community organization and supplemented some of the boxes that people were getting with like, collards, tomatoes, peaches, all free of cost. So we grow all our food and we donate it all to people. We don’t have any fences around our property, so neighbors can come and go as they please, taking what they want.

(Tyler, Farmer)

Tyler created a freely accessible space, open to the community, with a goal of providing food to those in need. Unlike grocery stores, excess food on Tyler’s farm is not thrown away, but given away, because profit is not the central goal.

### 3.3. Health Promotion

Improving community health was mentioned as a major goal and perhaps the greatest benefit of urban agriculture. Participants drew connections between using urban farming to increase the control and supply of food, enabling communities to improve their health. Regarding health promotion, most respondents discussed health education as a main focus of their work. For example, Rodney detailed how nutrition influences the quality of life and lifespan of low-resourced communities.

We’ve got to look to nutrition because it is the contributor to life opportunities…it’s a contributor to lifespan and quality of life, and in BIPOC (The term POC refers to “people of color,” individuals in the United States of African, Asian, Latin American, Middle Eastern, Arab, and/or Indigenous descent. BIPOC highlights specific intersections within POC groups, mainly the unique relationships Indigenous and Black (African American) people have within a U.S. context) communities that quality of life, that lifespan, that ability to get on is compromised in many ways because of nutritional issues.

(Rodney, Academic)

Several respondents mentioned that residents living in low-access areas may understand this relationship yet have little power to eat healthier food. Anita talks about integrating her farming practices with educational workshops on healthy eating, emphasizing how food serves as preventative medicine.

I started doing health and wellness education over 20 years ago, just teaching people cancer prevention…and I realized a lot of information we were given wasn’t really focused on food, and that’s the main cause…doing more research into food as medicine became a big focus for me.

(Anita, Farmer)

Anita notes that education is key to making best use of possible unfamiliar items grown on the farm, and using them in ways that work for families and create new habits:

Now a lot of people are getting those produce boxes and they are filled with things that people just are not familiar with using on a regular basis or at all. And I think that that’s the challenge, it’s the education…I’m even seeing people post like, “What is this? What am I supposed to do with this?” And I think that there’s a huge disconnect. I mean, it’s great that the food’s getting to the communities but it would waste and sit in a lot of refrigerators for months.

(Anita, Farmer)

Rosslyn also considers herself a farmer and food justice advocate, hoping to prevent food-related illnesses and disease.

I got people that I lost to diet-related illnesses, related to just being…Black in America. And I wanted to address it…[and] do what I could, whether that’s making produce more affordable or…being able to grow nutritious, organic food for people…that’s what keeps me going…I got family members to this day dealing with health challenges…and I know a bunch of kale can’t save the world, but maybe if they ate a little healthier, the longer they would be in my life.

(Rosslyn, Farmer)

Education was discussed as a collaborative goal with community members, to provide fresh food, and to help them achieve better health through education and healthy behaviors.

### 3.4. Environmental Sustainability

Finally, urban farming was noted as key to community sustainability and environmental sustainability. Respondents talked about sustainability in broad terms: growing food in responsible ways (e.g., damaging the natural resources as little as possible), leveraging the benefits of farming for environmental benefit. Lauren talks about being inspired by sustainable farming practices and integrating sustainability into how she grows food for the community:

I have a friend who was interested in permaculture which is a type of…farming that mimics how natural systems work. And so that got me thinking, if we could farm in a way that was sustainable, inspired by nature, and has reverence for nature, then maybe I could be into farming, because it wouldn’t just be depleting all of our natural resources. And then we started…the idea was what if we could actually grow food, like directly next to the people…cutting out travel and fossil fuels from the systems, because a lot of the studies that we had seen said that the biggest impact is how far our food traveled…that would be doing something amazing for your carbon footprint.

(Lauren, Farmer)

Similarly to farmer Jimmy, Lauren notes that the distance food needs to travel from producer to consumer is an environmental hazard. Therefore, many of the farmers in this study noted that growing food responsibly, locally, and in safe areas were of top concern. As Jimmy stated, “That’s what we need in Baltimore. Cleaner, greener foods, cleaner water, and clean air”. As described by several participants, creating local and sustainable agriculture was seen as a win-win in providing food to low-access areas, as well as creating awareness about other environmental issues often facing lower-income communities. Farmer Rich details one such example where a neighborhood is being mobilized around several environmental issues simultaneously:

The Cherry Hill Urban Farm is very close to the incinerator. It’s an impoverished, isolated part of the city. And [a worker] at Baltimore Compost Collective is engaging youth to build compost and collect food scraps from some of the wealthier neighborhoods that are paying for that service. And then they make compost with it. And it goes into…Filbert Street Garden. And everything over there in the Curtis Bay neighborhood is revolving around this political movement—this outcry, public outcry, to shut down this trash incinerator, for better air quality.

(Rich, Farmer)

Similarly, Phil expresses a motivation to continue food justice work because of his desire to keep others healthy, from an environmental perspective.

Where the garden is located is one of the most toxic communities in Baltimore City. It has two incinerators… [and] historically it’s been a dumping ground. When you burn trash, you create a chemical called carbon dioxide. When you bury trash, you create a chemical called methane, which causes $55 million in health damages for the residents of Baltimore City. They put these incinerators in poor neighborhoods but the wind doesn’t segregate or discriminate. So we’re all breathing in bad air…We know that the alternative to incineration is composting, so we’re…blessed to be a model for composting for Baltimore City.

(Phil, Farmer)

Phil collects waste in the community and composts it, and then uses the enriched soil as a fertilizer for the plants grown in his garden. His work is contributing to cleaner air in Baltimore City, which helps prevent those living near the incinerator from developing a number of lung problems, such as asthma or difficulty breathing.

### 3.5. Thematic Summary

In summary, several themes emerged from conversations with Baltimore community members, farmers, and academic researchers in response to questions about the city’s food inequities and urban agricultural practices. Participants drew connections between food access and systematic racism, noting subsequent issues in disparate pricing, quality, and distance in relation to accessing healthy food options. Urban agriculture was generally seen as underutilized; if community farming was further supported, it could provide community empowerment, health promotion, and education, and opportunities to integrate sustainable environmental practices into food distribution networks.

## 4. Discussion

Our findings indicate that urban agriculture has the potential to make healthy food more accessible and affordable for Baltimore city residents. Interviews with a diverse sample of community stakeholders indicate an alignment with a food sovereignty frame, that communities should control access to and production of their own food, and urban agriculture is one way to achieve that goal. Additionally, participants discussed that food deserts are the result of systemic discrimination and residential segregation. Food affordability was seen as a primary challenge to consistent food security, given the tradeoff between food pricing and quality. While programs such as SNAP were noted as alleviating some of this burden for very low-income families, they were deemed insufficient in meeting the larger needs of the community. Finally, a lack of transportation and distance to supermarkets were noted as a universal challenge to regular access to fresh food for Baltimore residents across income levels, making the role of local gardens and farms particularly salient. Likely due to the timing of data collection, existing challenges in food access were worsened by the pandemic.

Despite these challenges, providing opportunities for community control and management of local food sources via community-run gardens and farms can begin to redress the effects of segregation and provide opportunities for sustainability, community empowerment, and the provision of affordable and quality produce within proximity to residents. The primary theme, the importance of urban agriculture cooperative strategies in addressing food insecurity created by systemic racism, was noted throughout all the interviews by residents, academic scholars, food equity advisers, and urban farmers. In relation to the primary research question examining the role of urban farms in lower-income Baltimore communities, all stakeholders emphasized the role of farms in providing fresh, affordable, and high-quality food in low-access communities. Equally important, community gardens and farms were described as providing tangible support and assistance to the community by providing food, jobs, and a sense of belonging. Sites for urban agriculture were often regarded as community spaces, serving as places where residents could collect food, work, volunteer, and in some cases, gather to see their neighbors. In this way, urban agriculture can benefit nutritional health, as well as social and community health. 

The third theme, health promotion, was described as a holistic practice extending beyond simply eating the “right” foods. Several participants noted that “food is medicine” and that communities without sufficient access to healthy food remain sick, both physically and mentally. Finally, environmental sustainability through farming was noted as a way to alleviate the issues of citywide food insecurity and improve environmental conditions in low-income communities. 

Taken together, these findings indicate that urban agriculture, represented by farming cooperatives and community-run gardens, can produce healthy and affordable food that is community-controlled, potentially improving community health and well-being, and benefitting lower-income residents who may have lower access to traditional food retail. This work aligns with global and international human rights efforts to shift the production of food towards models that emphasize community control, food as a right rather than a commodity, and privilege access to food [23]. Additionally, since food and farming cooperatives are composed of diverse groups (e.g., farmers, volunteers, residents), urban agriculture and AFNs have the capacity to reduce the cost of food, increase availability, and build social capital, engagement, and community empowerment. Therefore, exploring new models of food production and distribution may address food deserts on a local/regional level, and disparate food access on a large scale.

In translating the implications of this work for future food policy, the United Nations Humans Rights Council (UNHRC) issued a 2008 report naming hunger as a barrier to health, concluding that “the right to food can only be realized where the conditions enabling food sovereignty are guaranteed” [73]. Therefore, policymakers should consider prioritizing food access through local agricultural production; doing so invests in local economies, protects local markets, reduces the cost of importing food, and ultimately improves health. In turn, communities able to build economic stability can and will invest in local infrastructure(s) that further supports community health (e.g., hospitals, schools) and environmental sustainability. 

Our interviews indicate no hesitation or shortage of farmers and residents wanting to pursue new food distribution models. Existing intergenerational programs, if supported at the city level, could expand to support younger farmers in learning agricultural methods. Farms could also become more efficient and diversify production with municipal support and plans designed to elevate and prioritize communities with the greatest need. Essentially, agriculture, planning, and health policy cannot be separated if food insecurity is to be seriously addressed.

### Limitations

As a largely exploratory effort, our study has several limitations. First, the COVID-19 pandemic limited participant recruitment efforts. This may have contributed to a smaller and less diverse sample of participants, limiting an in-depth understanding of urban agriculture in Baltimore. Also, as a case study focused on one city, the findings may not necessarily generalize to other contexts, both urban and non-urban. Future research may examine the role of urban agriculture in a variety of areas and diverse income-communities to determine the viability and feasibility of implementing models based on the concept of food sovereignty.

## 5. Conclusions

This study examined the role of urban agriculture in increasing access to food in Baltimore, MD. Findings suggest that beyond individual-level factors, neighborhood and structural factors play a significant role as well. The COVID-19 crisis highlighted existing gaps in how food is provided locally, and presented an opportunity to leverage community agricultural resources to build a more robust, resilient food economy that is representative of community diversity. Therefore, despite the seemingly entrenched nature of food deserts, evidence suggests that urban agriculture, particularly farms and gardens that are community-run and operated, provide numerous benefits beyond health, yielding potential improvements for community cohesiveness and environmental sustainability. Furthermore, our results show that community farmers are invested in the communities they serve, providing options for low-cost (and sometimes no-cost) fruits and vegetables, health education, and work opportunities.

The academic literature is severely limited in its characterization of solutions to low-food access areas, and the current study indicates that urban agriculture presents a reasonable and sustainable solution to rethinking and redesigning food distribution and food access in these communities. This study represents one of the few empirical studies examining urban agriculture as a potential contributor to food sovereignty. The lack of attention paid to AFNs generally, and community farms and gardens specifically, hinders a nuanced understanding of how community resources may shift systemic change in access to food. A broader conceptualization of food security and food access (one that includes AFNs) would allow support for the regional and national shifts needed to decentralize food production and provide healthy food where it is needed most.

## Data Availability

Not applicable.
